# Kennedy disease misdiagnosed as polymyositis: a case report

**DOI:** 10.1186/1756-0500-6-389

**Published:** 2013-09-28

**Authors:** Grigor M Harutunian, Said R Beydoun, Richard A Rison

**Affiliations:** 1 University of Southern California, Keck School of Medicine, Los Angeles County Medical Center, 1520 San Pablo Street Suite 3000, Los Angeles, CA 90033, USA; 2 Medical Director PIH Health Stroke Program, University of Southern California Keck School of Medicine, Los Angeles County Medical Center, 12401 Washington Blvd. Whittier, California 90602, USA

## Abstract

**Background:**

Polymyositis is an immune-mediated myopathy with clinical features of proximal muscle weakness. Dysphagia and neck flexor weakness can develop along with respiratory muscle weakness as the disease progresses. Kennedy disease or X-linked spinobulbar muscular atrophy is a rare X-linked recessive disorder with clinical features of slowly progressive atrophy and weakness of limb and bulbar muscles. These two disorders may have overlapping clinical manifestations.

**Case presentation:**

We present the case of a 52-year-old Filipino man with chronic weakness involving his proximal muscle groups who carried the diagnosis of polymyositis and was refractory to multiple immunomodulatory therapies. Further neurologic examination and history taking along with selective serologic and electrodiagnostic studies instead confirmed the diagnosis of Kennedy disease.

**Conclusions:**

Distinction between polymyositis and Kennedy disease may be difficult given the potential overlapping clinical manifestations. However, with careful neurological history taking, examination, and selective serologic plus electrodiagnostic investigations the correct diagnosis may be made, thus sparing the patient ineffective therapy. One must always be sure of the diagnosis of polymyositis before it’s classified as refractory.

## Background

Polymyositis (PM) and dermatomyositis (DM) are T cell immune-mediated inflammatory myopathies affecting females slightly more than males commonly during the 2nd to 4th decades. DM typically presents similarly to PM with the exception of the skin findings. Muscle pain and tenderness are common and usually more prominent in the arms. Onset of symptoms is subacute or insidious. With disease progression, dysphagia and neck flexor weakness can develop along with respiratory muscle weakness. Myositis is nonspecific and can be a manifestation of a number of other related conditions [1-4].

Kennedy disease or X-linked spinobulbar muscular atrophy is a rare X-linked recessive disorder caused by CAG trinucleotide repeat expansion in the androgen receptor gene on chromosome Xq11-12. Slowly progressive atrophy and weakness of limb and bulbar muscles occur beginning in the 3rd to 5th decades. Muscle cramps, fasciculations, and characteristic perioral myokymia may precede the onset of weakness and wasting. Proximal hip and shoulder girdle weakness dominates with most affected individuals having difficulty walking up stairs after 1 to 2 decades of symptoms. Affected individuals also show signs of mild androgen insensitivity manifesting as gynecomastia, testicular atrophy, and reduced fertility, but sexual differentiation and development of secondary sexual characteristics are normal [1,2,5,6].

## Case presentation

The patient is a 52-year-old married Filipino man who developed weakness greater than 10 years ago. Our first encounter with the patient was in May 2011 when he was referred to our infusion center for intravenous immunoglobulin (IVIG) treatment for presumed PM. He first noticed difficulty with ambulating especially climbing stairs and standing up from a seated position. In addition, he had difficulty reaching for items from cabinets, complained of some difficulty with swallowing liquids, and stated that he had decreased libido and significant difficulty achieving an erection and lacked any active sexual life. He denied any associated pain, sensory symptoms, cardiac involvement, or muscle hypertrophy. Interestingly, the patient noted an older deceased brother who had similar symptoms along with a maternal uncle and maternal cousins with similar symptoms.

The patient carried a diagnosis of polymyositis dating back to 2000. At that time investigations included an elevated creatine kinase (CK) of 1522 IU/L, a magnetic resonance imaging (MRI) of the leg showing atrophy and increased signal on short TI inversion recovery (STIR) images, but no electromyogram, nerve conduction studies, or muscle biopsy. Given these findings along with his clinical picture, he was however diagnosed with polymyositis and treated with a trial of high-dose prednisone, which did not improve his clinical symptoms but did improve his CK levels according to his wife. He was also treated with various immunosuppressive medications, which he failed, including methotrexate, azathioprine, cyclosporine, and mycophenolate.

A muscle biopsy performed in 2007 showed end-stage muscle atrophy with severe myofiber atrophy and fatty replacement of muscle. A follow-up MRI of the right thigh in January 2011 showed muscle atrophy and edema in the right thigh. A formal swallow evaluation in April 2011 showed trace aspiration with a large bolus of thin liquids with residual in the valleculae. He then received IVIG treatment at a dose of 2 g/kg on 5/31/2011, divided over 3 days. The patient indicated no improvement similar to the prior treatments with prednisone and multiple immunosuppressive medications.

Upon examination of the patient, gynecomastia was noted. The neurological exam was positive for lower facial weakness with evidence of myokymia in the chin muscles and tongue atrophy with fasciculations. Strength testing demonstrated proximal weakness in the upper and lower extremities with trace to absent reflexes.

An electrodiagnostic study showed evidence of diffuse chronic lower motor neuron changes with active and chronic denervation potentials seen in cervical, lumbosacral, and cranial-innervated myotomes. Based on the history, examination findings, electrodiagnostic results, and X-linked inheritance pattern, Kennedy disease was highly suspected. The genetic test for Kennedy disease was sent and came back positive, showing that in 1 allele there was expansion in the number of CAG repeats, 49 repeats, consistent with full mutation of the gene thereby confirming the suspected diagnosis of Kennedy disease. The patient was without offspring. He accepted the diagnosis, and was grateful for knowing the correct diagnosis and not needing inappropriate therapy. He was counseled about the diagnosis, mode of inheritance, course, and prognosis.

## Discussion

Our patient had carried an incorrect diagnosis for more than 10 years based on the clinical findings of progressive proximal weakness with an associated elevated CK level and MRI evidence of thigh muscle atrophy and increased signal, which lead to multiple failed treatment trials with a variety of immunosuppressive drugs including a trial of IVIG.

Electrodiagnostic findings in PM usually demonstrate normal motor and sensory nerve conduction studies with needle electromyography (EMG) showing a myogenic pattern and increased spontaneous activity. Muscle biopsies demonstrate endomysial mononuclear inflammatory infiltrate with muscle fiber necrosis [2-4]. Dissimilarly, electrodiagnostic findings in Kennedy disease commonly demonstrate normal motor studies but most patients have low amplitude or absent sensory nerve action potentials with needle EMG showing neurogenic changes in affected muscles. Facial muscles may show grouped repetitive motor unit discharges upon mild activation. Because these discharges occur with mild voluntary contraction rather than spontaneously, they are distinguished from myokymic or neuromyotonic discharges and are characteristic of Kennedy disease. Muscle biopsies demonstrate nuclear inclusion bodies containing the mutated truncated androgen receptor frequently found within motor neurons in the brainstem and spinal cord [2,5,6].

Kennedy disease and PM can have elevated CK levels and progressive proximal weakness in common but there are distinguishing characteristics. “Kennedy’s disease should be suspected in any patient with proximal and bulbar weakness, a positive family history, facial fasciculations, and/or gynecomastia whose electrodiagnostic studies show abnormal sensory findings in addition to the typical widespread neuronopathic pattern on needle EMG” [1]. “Polymyositis should be suspected in a patient presenting with subacute to chronic symmetric proximal muscle weakness and occasional bulbar signs with sparing of the facial muscles whose electrodiagnostic studies show normal motor and sensory nerve conduction studies with a myogenic pattern seen on needle EMG” [1]. Additionally, PM can occur in association with autoimmune disease and in the setting of malignancy [2-4].

Unfortunately in our patient’s case, an EMG/nerve conduction study and muscle biopsy were not performed until at least 7 years after the onset of his symptoms. If these studies had been performed earlier in the course of the patient’s investigations, then this information along with the fact that there was no clinical response to therapy should have called into question the initial diagnosis of PM much sooner thereby sparing the patient ineffective treatment.

## Conclusions

Distinguishing between PM and Kennedy disease can be difficult. A thorough evaluation of the clinical history including family history along with a careful detailed neurological and electrophysiological examination plus genetic testing usually helps direct the physician towards the correct diagnosis and thus may spare the patient ineffective treatment. One must always be sure of the diagnosis of polymyositis before it’s classified as refractory.

## Consent

Written informed consent was obtained from the patient for publication of this case report and accompanying images. A copy of the written consent is available for review by the Editor-in-Chief of this journal.

## Competing interests

The authors declare that they have no competing interests.

## Authors’ contributions

GMH and SRB were both involved in clinical diagnostic evaluation and management. GMH and SRB performed the electrodiagnostic studies. GMH generated the first draft of the manuscript. SRB and RAR reviewed the manuscript. RAR revised and edited the manuscript using an additional literature search. SRB and RAR were responsible for the intellectual content of the paper. All authors participated in and provided significant contributions in writing the manuscript. All authors read and approved the final manuscript.

## Authors’ information

GMH is a neurophysiology fellow at the University of Southern California-Keck School of Medicine-Los Angeles County Medical Center. SRB is a professor of neurology at the University of Southern California-Keck School of Medicine-Los Angeles County Medical Center. SRB is Director of the University of Southern California Neuromuscular Program, a Fellow of the American Academy of Neurology and the American Association of Neuromuscular and Electrodiagnostic Medicine, and is board certified by the American Board of Psychiatry and Neurology in Neurology, Clinical Neurophysiology, Pain Medicine, and Neuromuscular Medicine. SRB is also board certified by the American Board of Electrodiagnostic Medicine in Electrodiagnostic Medicine. SRB is a member of the advisory board and the scientific committee of the Myasthenia Gravis Foundation of California. RAR is a Deputy Editor for the *Journal of Medical Case Reports*, an Associate Neurology Editor for *Grand Rounds* and *WebmedCentral*, and a Section Editor for *BMC Research Notes*. RAR practices general neurology at Neurology Consultants Medical Group, serves as Medical Director of the PIH Health Stroke Program, is a Clinical Assistant Professor of Neurology at the University of Southern California-Keck School of Medicine-Los Angeles County Medical Center, and is a Fellow of the American Association of Neuromuscular and Electrodiagnostic Medicine. RAR is board certified by the American Board of Psychiatry and Neurology in Neurology and Vascular Neurology, and Neurocritical care and Neuroimaging by the United Council of Neurologic Subspeciatlies. RAR is also board certified by the American Board of Electrodiagnostic Medicine in Electrodiagnostic Medicine. RAR is a former president of the Los Angeles Neurological Society.
